# Supplementary figures and images for: Health system response to COVID-19 among primary health care units in Ethiopia: A qualitative study
Source: PLoS One. 2023 Feb 10;18(2):e0281628. doi: 10.1371/journal.pone.0281628 (PMC9916627; doi:10.1371/journal.pone.0281628)

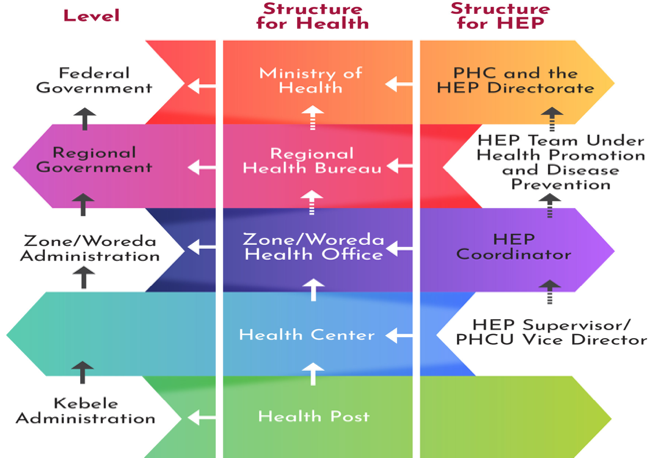


Source: MOH, Roadmap for Optimizing the Ethiopian Health Extension Program 2020-2035. July 2020, Addis Ababa.

Supplement: S1 Fig — (DOCX) [file pone.0281628.s001.docx]
