# Supplementary material for: Health system response to COVID-19 among primary health care units in Ethiopia: A qualitative study
Source: PLoS One. 2023 Feb 10;18(2):e0281628. doi: 10.1371/journal.pone.0281628 (PMC9916627; doi:10.1371/journal.pone.0281628)
Supplement: Attachment — Submitted filename: PONE-D-22-03390_reviewer.pdf [file pone.0281628.s002.pdf]

PLOS ONE

Health system response to COVID-19 among primary health care units in Ethiopia: A qualitative study

--Manuscript Draft--

Manuscript Number:	PONE-D-22-03390
Article Type:	Research Article
Full Title:	Health system response to COVID-19 among primary health care units in Ethiopia: A qualitative study
Short Title:	Health system response to COVID-19 among primary health care units in Ethiopia: A qualitative study
Corresponding Author:	Getnet Mitike Kassie, M.D. Ph.D.<br>International Institute for Primary Health Care - Ethiopia<br>Addis Ababa, ETHIOPIA
Keywords:	COVID-response, PPE, supplies, contact-tracing, multisectoral, pandemic, Rapid-response-team
Abstract:	Objective: The aim of the study was to explore the response of primary health care units to COVID-19 pandemic by the Ethiopian health system and the coordinating bodies.<br>Methods: We conducted a qualitative study with descriptive phenomenological design using 59 key informants that were purposively selected. The interviews included leaders across Ethiopia’s 10 regions and 2 administrative cities. Data were collected using a semi-structured interview guide that was translated into a local language. The interviews were conducted in person or by phone. Extensive Coding and categorizing led to the development of themes and subthemes. Data were analysed using thematic analysis.<br>Results: Local administrators across different levels took the lead in responding to COVID-19 by organizing multisectoral planning and monitoring committees. Health leaders reacted to the demand for an expanded workforce by reassigning health professionals to COVID surveillance and case management activities, adding COVID-related responsibilities to their workloads, temporarily blocking leave, and hiring new staff on contractual basis. Training was prioritized for frontline workers: members of rapid response teams, laboratory technicians, clinicians assigned to treatment centers, and health extension workers. COVID supplies and equipment, particularly personal protective equipment, were difficult to obtain at the beginning of the pandemic. Health officials used a variety of resourceful means to equip and protect staff, but the quantity fell short of their needs. Local health structures used broadcast media, print materials, and house-to-house education to raise community awareness about COVID-19. Rapid response teams took the lead in case investigation, contact tracing, and sample collection. Case management shifted to home-based isolation and care for mild cases as the number of infections increased and space became limited. However, essential health services were neglected at the beginning of the pandemic while local multisectoral response dropped as it progressed.<br>Conclusion: Local government authorities and health systems across Ethiopia waged an early response to the pandemic, drawing on multisectoral support and directing human, material, and financial resources toward the effort. But, the intensity of the response waned and essential services began suffering as the pandemic progressed. There is a need to invest in the basics of the health system – health workers, supplies and equipment, and infrastructure – as well as specific interventions to shore up the multi-faceted response against COVID-19.
Order of Authors:	Getnet Mitike Kassie, M.D. Ph.D.
	Frehiwot Nigatu, MD, MPH
	Eskinder Wolka, PhD
	Atkure Defar, PhD
	Masresha Tessema, PhD

Powered by Editorial Manager® and ProduXion Manager® from Aries Systems Corporation

	Tezita Negussie, MSW, MPH
**Additional Information:**	
**Question**	**Response**
**Financial Disclosure**<br><br>Enter a financial disclosure statement that describes the sources of funding for the work included in this submission. Review the submission guidelines for detailed requirements. View published research articles from *PLOS ONE* for specific examples.<br><br>This statement is required for submission and **will appear in the published article** if the submission is accepted. Please make sure it is accurate.<br><br>**Unfunded studies**<br>Enter: *The author(s) received no specific funding for this work.*<br><br>**Funded studies**<br>Enter a statement with the following details:<br>• Initials of the authors who received each award<br>• Grant numbers awarded to each author<br>• The full name of each funder<br>• URL of each funder website<br>• Did the sponsors or funders play any role in the study design, data collection and analysis, decision to publish, or preparation of the manuscript?<br>• **NO** - Include this sentence at the end of your statement: *The funders had no role in study design, data collection and analysis, decision to publish, or preparation of the manuscript.*<br>• **YES** - Specify the role(s) played.<br><br>* typeset	This study was funded by the Bill and Melinda Gates Foundation.<br>Disclaimer: The contents of this report are solely the responsibility of the authors and do not necessarily represent the official views of the Bill and Melinda Gates Foundation The funding is through the International Institute for Primary Health Care-Ethiopia. The funders had no role in the study design, data collection and analysis, decision to publish or preparation of the manuscript
**Competing Interests**<br><br>Use the instructions below to enter a competing interest statement for this submission. On behalf of all authors, disclose any competing interests that could be perceived to bias this work—acknowledging all financial support and any other relevant financial or non-financial competing interests.	The authors have declared that no competing interests exist

Powered by Editorial Manager® and ProduXion Manager® from Aries Systems Corporation

This statement is required for submission and **will appear in the published article** if the submission is accepted. Please make sure it is accurate and that any funding sources listed in your Funding Information later in the submission form are also declared in your Financial Disclosure statement.<br><br>View published research articles from *PLOS ONE* for specific examples.<br><br>**NO authors have competing interests**<br><br>Enter: *The authors have declared that no competing interests exist.*<br><br>**Authors with competing interests**<br><br>Enter competing interest details beginning with this statement:<br><br>*I have read the journal's policy and the authors of this manuscript have the following competing interests: [insert competing interests here]*<br><br>* typeset	
**Ethics Statement**<br><br>Enter an ethics statement for this submission. This statement is required if the study involved:<br><br>• Human participants<br>• Human specimens or tissue<br>• Vertebrate animals or cephalopods<br>• Vertebrate embryos or tissues<br>• Field research<br><br>Write "N/A" if the submission does not require an ethics statement.<br><br>General guidance is provided below. Consult the submission guidelines for detailed instructions. **Make sure that all**	Approved by the Ethiopian Public Health Institute – Institutional Review Board (EPHI IRB # 280-2020). The IRB approved the informed verbal consent to minimise contact through paper/pen exchange considering the COVID-19 transmission

Powered by Editorial Manager® and ProduXion Manager® from Aries Systems Corporation

**information entered here is included in the Methods section of the manuscript.**<br><br>**Format for specific study types**<br><br>**Human Subject Research (involving human participants and/or tissue)**<br>• Give the name of the institutional review board or ethics committee that approved the study<br>• Include the approval number and/or a statement indicating approval of this research<br>• Indicate the form of consent obtained (written/oral) or the reason that consent was not obtained (e.g. the data were analyzed anonymously)<br><br>**Animal Research (involving vertebrate animals, embryos or tissues)**<br>• Provide the name of the Institutional Animal Care and Use Committee (IACUC) or other relevant ethics board that reviewed the study protocol, and indicate whether they approved this research or granted a formal waiver of ethical approval<br>• Include an approval number if one was obtained<br>• If the study involved *non-human primates*, add *additional details* about animal welfare and steps taken to ameliorate suffering<br>• If anesthesia, euthanasia, or any kind of animal sacrifice is part of the study, include briefly which substances and/or methods were applied<br><br>**Field Research**<br><br>Include the following details if this study involves the collection of plant, animal, or other materials from a natural setting:<br>• Field permit number<br>• Name of the institution or relevant body that granted permission	
**Data Availability**<br><br>Authors are required to make all data underlying the findings described fully available, without restriction, and from the time of publication. PLOS allows rare exceptions to address legal and ethical	Yes - all data are fully available without restriction

concerns. See the PLOS Data Policy and FAQ for detailed information.<br><br>A Data Availability Statement describing where the data can be found is required at submission. Your answers to this question constitute the Data Availability Statement and **will be published in the article**, if accepted.<br><br>**Important:** Stating 'data available on request from the author' is not sufficient. If your data are only available upon request, select 'No' for the first question and explain your exceptional situation in the text box.<br><br>Do the authors confirm that all data underlying the findings described in their manuscript are fully available without restriction?	
**Describe where the data may be found in full sentences. If you are copying our sample text, replace any instances of XXX with the appropriate details.**<br><br>• If the data are **held or will be held in a public repository**, include URLs, accession numbers or DOIs. If this information will only be available after acceptance, indicate this by ticking the box below. For example: *All XXX files are available from the XXX database (accession number(s) XXX, XXX.*).<br>• If the data are all contained **within the manuscript and/or Supporting Information files**, enter the following: *All relevant data are within the manuscript and its Supporting Information files.*<br>• If neither of these applies but you are able to provide **details of access elsewhere**, with or without limitations, please do so. For example:<br><br>*Data cannot be shared publicly because of [XXX]. Data are available from the XXX Institutional Data Access / Ethics Committee (contact via XXX) for researchers who meet the criteria for access to confidential data.*	No

The data underlying the results presented in the study are available from (include the name of the third party and contact information or URL).<br>• This text is appropriate if the data are owned by a third party and authors do not have permission to share the data.<br><br>* typeset	
Additional data availability information:	Tick here if the URLs/accession numbers/DOIs will be available only after acceptance of the manuscript for publication so that we can ensure their inclusion before publication.

Powered by Editorial Manager® and ProduXion Manager® from Aries Systems Corporation

Manuscript

Click here to access/download;Manuscript;Manus_Covid response_v01_02_02-22.doc

Health system response to COVID-19 among primary health care units in Ethiopia: A qualitative study

Getnet Mitike* (MD, PhD)[1], Frehiwot Nigatu (MD, MPH)[1], Eskinder Wolka (PhD),[1]Atkure Defar (PhD)[2], Masresha Tessema (PhD)[2], Tezita Nigussie (MSW, MPH)[1]

[1]International Institute for Primary Health Care-Ethiopia; [2] Ethiopian Public Health Institute

* Corresponding author (email address: getnet.mitike@iifphc.org)

Abstract

Introduction: There was limited data on the experiences and roles of sub-national health systems in the response against COVID-19~~, particularly~~ in Ethiopia. This study explored how sub-national primary health care units in Ethiopia responded to COVID-19 during the first 6 months of ~~experiencing the~~ pandemic.

Objective: The aim of the study was to explore the response of primary health care units to COVID-19 pandemic by the Ethiopian health system and the coordinating bodies.

Methods: We conducted a qualitative study with descriptive phenomenological design using 59 key informants that were purposively selected. The interviews included leaders across Ethiopia's 10 regions and 2 administrative cities. Data were collected using a semi-structured interview guide that was translated into ~~a~~ local language. The interviews were conducted in person or by phone. Extensive Coding and categorizing led to the development of themes and subthemes. Data were analysed using thematic analysis.

Results: Local administrators across different levels took the lead in responding to COVID-19 by organizing multisectoral planning and monitoring committees. Health leaders reacted to the demand for an expanded workforce by reassigning health professionals to COVID surveillance and case management activities, adding COVID-related responsibilities to their workloads, temporarily blocking leave, and hiring new staff on contractual basis. Training was prioritized for frontline workers: members of rapid response teams, laboratory technicians, clinicians assigned to treatment centers, and health extension workers. COVID supplies and equipment, particularly personal protective equipment, were difficult to obtain at the beginning of the pandemic. Health officials used a variety of resourceful means to equip and protect staff, but the quantity fell short of their needs. Local health structures used broadcast media, print materials, and house-to-house education to raise community awareness about COVID-19. Rapid response teams took the lead in case investigation, contact tracing, and sample collection. Case management shifted to home-based isolation and care for mild cases as the number of infections increased and space became limited. However, essential health services were neglected at the beginning of the pandemic while local multisectoral response dropped as it progressed.

Conclusion: Local government authorities and health systems across Ethiopia waged an early response to the pandemic, drawing on multisectoral support and directing human, material, and financial resources toward the effort. But, the intensity of the response waned and essential services began suffering as the pandemic progressed. There is a need to invest in the basics of the health system – health workers, supplies and equipment, and infrastructure – as well as specific interventions to shore up the multi-faceted response against COVID-19.

Key words: COVID-response, PPE, supplies, contact-tracing, multisectoral, pandemic, Rapid-response-team

Introduction

After the World Health Organization (WHO) declared Public Health Emergency of International Concern in Early January 2020, COVID-19 became a global threat and a pandemic in March 2020 (1). The COVID pandemic has so far affected and killed millions of people globally (2). From the beginning, COVID-19 caught the attention of governments for fear of its multifaceted impacts on populations, health systems, economies, social relationships, and political repercussions. In Africa, early projections and learning from the impacts Ebola outbreak, expected calamities from COVID-19 were concerning, considering the fragile health systems, weak health security capacity and poor economies (3-8).

In Ethiopia, the first case of COVID-19 was detected on March 14, 2020. In April 2020, the government issued a five-month state of emergency that closed schools and universities, banned gatherings of more than four people, ordered transportation service providers to reduce their passenger loads, prohibited sports activities, and mandated the wearing of masks in public (9-10). In collaboration with the WHO and other partners, the government also began assessing and equipping public and private laboratories to expand testing for COVID-19 (11).

Much of the early published research work in Africa at the time examined national responses based on document reviews (12-21), and the empirical studies, particularly from Ethiopia, focused on knowledge, attitude and practices toward coronavirus prevention measures among community members and health professionals (22-35). Furthermore, approaches and processes to systematically mount the response against COVID-19 was largely unknown particularly at subnational level where most of the preventive activities are taking place. In a decentralized system such as Ethiopia exploring the response including organizational approaches towards COVID-19 at subnational (regional) and primary health care levels will offer a better picture of the process, measures undertaken to control and prevent the spread of coronavirus. This study is therefore to understand the process and learn from the response

towards COVID-19 pandemic at subnational level in Ethiopia considering the first 6 months of the pandemic.

Materials and Methods

Study setting: The study was conducted in September and October of 2020 in Ethiopia’s ten regions (Afar, Amhara, Benishangul-Gumuz, Gambella, Harari, Oromia, Sidama, Somali, Southern Nations Nationalities and Peoples, and Tigray) and two administrative cities (Addis Ababa and Dire Dawa). The regions and administrative cities are divided into zones or sub cities, zones are further divided into woredas (districts), and each woreda consists of kebeles, which are the lowest administrative units. The health system is organised into three tier system represented by primary health care units (primary level), general hospital (secondary level) and tertiary referral hospital (tertiary level) each with catchment populations. A Primary Health Care Unit (PHCU) consists of a health center, five health posts and a primary hospital (36). Health authorities are present at every administrative level and serve different functions. Regional health bureaus work in conjunction with the Ministry of Health (MOH) to set targets and develop plans for lower levels; zonal health departments implement or enforce polices and plans developed by the regional health bureaus; and woreda health offices plan, implement, and evaluate activities at the woreda level. The woreda health offices also manage the health posts and health centers that function as the core of Ethiopia’s primary health care (36).

Study design: We employed descriptive qualitative (phenomenological) design to explore the Ethiopian health system’s response to COVID-19 in the first six months of the pandemic.

Selection of participants and sample size: We used purposive sampling to select a total of 59 participants. Key informants were chosen among health professionals who were actively engaged in the COVID response across the country. Active engagement included direct participation in decision making pertaining to prevention and control and provision of services to patients affected by

coronavirus. From each region and city administration, we selected one person from a regional health bureau, two persons from zonal/woreda health offices, and two from health facilities. The criteria for selection were completion of post-secondary education, current employment in the public health system and involvement in COVID response. In our sampling strategy we covered all regions (subnational) of the country and followed the decentralization system with the aim of obtaining some kind of representation of the administrative and service provision capacities at various levels. This was important as there were differences in the capacities of the regions with regards to human resource, distribution of facilities and modalities of health services provision.

Data Collection and Analysis: We recruited and trained 12 data collectors (interviewers) and 12 supervisors. Both groups had graduate level education with some qualitative research experience. The supervisors made contacts and arranged meetings, communicated with the principal investigator (PI) and checked voice records, transcripts and translated versions of the interviews. The interviewers on the other hand conducted the interviews, transcribed and translated them to English. Because of government restrictions on assembly and travel, we held a training for data collectors and supervisors remotely via Google Meet for half a day. The data collectors conducted interviews in person or by phone using a semi-structured interview guide that was translated into Amharic, the national language. The data collectors digitally recorded the interviews and translated and transcribed the responses into English. The supervisors met (in person or via phone) with the data collectors each day to review the transcripts and discuss ways to improve the quality of data collection. The supervisors provided feedback and support in consultation with the PI.

Two experts with previous experience and training in qualitative methods read the transcripts independently. The PI is trained in qualitative methods at graduate level and has co-authored papers in peer reviewed journals and the other has graduate level training in social work and public health. The

transcripts were coded and a code book was developed. The codes were revised several times over the course of the analysis between the PI and the co-investigator. From the codes themes and sub themes were developed.

Validity and reliability of qualitative research is measured through trustworthiness which in turn includes credibility, confirmability, transferability and dependability (37). To ensure the credibility of our findings, we recruited data collectors and supervisors who were actively working in the respective regions and who were familiar with their respective study areas and health systems. The data collectors and supervisors also received training on the purpose, methods, and tools of the study. We accounted for confirmability by capturing participants' responses through recordings and transcriptions that were checked by supervisors and read several times by investigators. The study addressed transferability by including all regions of the country as the infrastructures and context are at varying levels. We also validated the semi-structured guides through discussion with the interviewers and supervisors. For ensuring dependability, the guides for the interview include interventions and strategies recommended by the WHO and the country context. The methods and procedures applied are consistent with other authors that conducted similar studies.

The proposal was approved by the Institutional Review Board of Ethiopian Public Health Institute (EPHI) (# 280-2020). All study participants provided informed verbal consent to be interviewed. Verbal consent was used to minimise contact through paper exchange considering COVID-19 transmission.

Results

Study Participants: This qualitative analysis was made using the transcripts of fifty-nine (59) health care professionals participated in the study. Eighty-nine percent were male. Participants ranged substantially in age, with a mean age of 35. Forty four percent of the health facilities were well

represented in the sample. Participants were in senior leadership or management positions, but, on average, had been on the job less than five years (Table 1).

Table 1 Characteristics of study participants in selected Primary Health Care Units, Ethiopia, 2020*

Characteristics	Number (percent)
Age *(n=58)*	
Mean	35
Range	26-58
Sex *(n=59)*	
Male	53 (89.8%)
Female	6 (10.2%)
Highest level of education *(n=59)*	
BSc	28 (47.5%)
MA/MSc/MPH	25 (42.4%)
MD	4 (6.8%)
Other	2 (3.4%)
Profession *(n=59)*	
Public health	26 (44.1%)
Nursing	10 (16.9%)
Health Officer	10 (16.9%)
Medicine	4 (6.8%)
Environmental health	3 (5.1%)
Midwifery	2 (3.4%)
Other	4 (6.8%)
Level *(n=59)*	
Health facility	26 (44.1%)
Regional health bureau	16 (27.1%)
Zonal health department	8 (13.6%)
Woreda health office	8 (13.6%)
Unknown	1 (1.7%)
Position *(n=58)*	
Head of zonal health department, woreda health office or health facility	26 (44.8%)
Head of division, team, or service	16 (27.6%)
Public health emergency manager	7 (12.1%)
Deputy head of regional health bureau	5 (8.6%)
Other	4 (6.9%)
Number of years in position *(n=58)*	
Mean	4
Range	2 months to 14 years
Total years of professional experience *(n=59)*	
Mean	12
Range	2-40

* The *n* varies for some questions due to non-response. Overall, N=59.

The results ~~are~~ categorized into 5 themes and subthemes: 1) Early Activities: Containment, Coordination, and Incidence Management; 2) Operational Support and Logistics (have three sub themes; *Health workforce, Training, COVID-19 supplies and equipment*); 3) Risk Communication and

Community Engagement; 4) Surveillance, Rapid Response Teams, and Case Investigation; 5) Case Management and Infection Prevention and Control (two sub themes: *Case management and risk infection prevention and control*).

1. Early activities: Containment, Coordination and Incidence Management

Preparations for containment measures were implemented in a few regions of Ethiopia before the first cases of SARS-COV-2 was reported on March 14, 2020. Some of measures included screening at points of entry such as airports and cross border sites. Accordingly, regional and zonal leaders instituted new entrant's screenings at the points of entry. For example, regional leaders in Afar established entrance screening at a major border crossing with Djibouti and airport screening was set up in Amhara region. These activities were instituted based on the WHO declaration and the alert letter from the Ethiopian Public Health Institute. Containment measures were based on the guidelines and case definition of the WHO which was communicated to the subregional levels.

After the first cases of COVID-19 were confirmed, administrative bodies – from regional councils to lowest administrative units (kebeles) – formed coordination committees. The heads of administrative bodies presided over these coordinating bodies (multisectoral committees) that planned, organized, and monitored local responses. Different sectors were represented on these committees, including health; education; finance; defence; water, irrigation and electricity; and trade.

Sub-committees were also formed to implement specific tasks. Key informants frequently cited the finance or resource mobilization sub-committee, which was responsible for obtaining support - in cash and kind - from local governments, non-governmental organizations, community members. The coordination committees also raised and directed resources to the most vulnerable members of their communities. *"Another committee established by the mayor was the resource mobilization committee, which includes*

the finance and health sectors, and coordinates resource mobilization to purchase personal protective equipment like face masks and provides support for community members who are seriously affected." (Sidama, Zonal Health Department).

The coordinating committees met regularly at the beginning of the pandemic to evaluate their work and plan next steps. As the months progressed, however, key informants from several regions noted a decline in commitment as other issues gained priority or as panic over the virus subsided.

Surveillance was a key focus of incidence management teams across regions, particularly at the woreda level. At higher levels, the initial focus was on operations: assessing the extent of need for and setting up quarantine, isolation, and treatment centers; customizing or developing guidelines for health workers; and expanding screenings at major entry points. Health centers organized rapid response teams to conduct case finding and contact tracing in their communities.

"Before health centers began any activity, they organized committees. The committees consisted of seven or eight people, including health professionals and supportive staff. After establishing the committees, we divided tasks such as providing community awareness and availing supplies. … if you look at the report there were committees both in 2012 and 2013 [Ethiopian calendar] and those committees had sub-committees that provided support for those in need at the community level, which included distributing sanitizers, face masks, food, and money. In addition, there were also committees working on providing orientation and education for health professionals." (Oromia, Health Facility 1).

2. Operational Support and Logistics

Health workforce: As most key informants described, health leaders responded to the heightened demand for healthcare workers in two ways: 1) by redeploying or adding responsibilities to the existing workforce, and 2) by hiring new workers. Often, authorities adopted the first approach. Health professionals from hospitals and health centers were redeployed to isolation and treatment centers, rapid response teams (RRT), and care teams for patients in home-based isolation. They were also asked to serve in woreda health offices and regional health bureaus as members of planning and coordination teams.

Health extension workers, who were already engaged in community education, mobilization and prevention activities, added COVID-related tasks to their day-to-day activities by leading community-based education, screening efforts and assisting rapid response teams with contact tracing. The added responsibilities created pushback: "*Most services, including community services and COVID, rest on the shoulders of the health extension workers. That is why they complained about the work burden.*" (Sidama Health Facility 2). The redeployment of health professionals affected the delivery of essential health services. According to a regional health bureau official in Benishangul-Gumuz, *"As soon as COVID-19 occurred, by providing training, we mobilized the health workforce that was previously providing essential health services.*" New staff were rarely hired to replace the ones who were assigned to work on COVID activities and, as a result, routine services were interrupted or suffered in quality in the first three months of the pandemic.

To maintain the capacity of the existing workforce, the health system instituted a no leave policy that denied health workers their annual leave. "*Annual leave was prohibited to all professionals because if all professionals were not working together, the pandemic could affect many people.*" (Harari, Regional Health

Bureau). However, during the state of emergency, certain categories of workers – pregnant and lactating women and providers with chronic conditions – were ordered to stay home.

Additionally, health authorities in Addis Ababa, Afar, Benishangul-Gumuz, Oromia, Southern Nations, Nationalities and Peoples' (SNNP), and Tigray regions hired new staff. They received money from the regional health bureau, regional government, or Ministry of Health to increase their health workforce, but often temporarily. The new hires were largely professionals - medical doctors, health officers, environmental health experts, laboratory technicians, and nurses. Despite the investment in health professionals, key informants in Addis Ababa complained of a severe shortage of urban health extension workers in the capital.

Training: Training for the health workers focused on key pillars of COVID-19 response - surveillance (specifically, case investigation by RRTs), infection prevention and control, community education, and case management. Health authorities relied on the train-the-trainer model to transfer skills and information from regional health bureaus down to health centers and health posts.

Theoretically, teams of content experts in regional health bureaus were expected to train their counterparts in zonal health departments who, in turn, would train selected staff at woreda health offices and health centers. Trained staff from woreda health offices and health centers were responsible for training colleagues in their institutions. More often, however, regional health authorities suspended the linear train-the-trainer model and directly offered training to staff at lower levels. Regional health staff trained health professionals involved in highly technical or advanced care, such as laboratory technicians and clinicians assigned to isolation and treatment centers. In Tigray and Benishangul-Gumuz, regional health staff trained the rapid response team at a woreda health office. It was not clear why the train-the-trainer model was suspended in some cases and the impact of this decision on the efficacy of trainings was unknown.

Content specific trainings were organized for RRTs, staff working at quarantine, isolation and treatment centers, laboratory technicians, and health extension workers. In Amhara, informants mentioned that influential community members and health professionals from the private sector were also invited to participate in trainings, particularly on infection prevention and control. The duration of trainings differed based on the topic. Trainings were held online, in venues that were large enough to meet physical distancing requirements, or on-site.

In most regions, trainings organized by regional or zonal offices were supported by partner institutions. Wolaita Sodo University, Mekelle University, and Bahir Dar University, for example, assisted with trainings in SNNP, Tigray, and Amhara, respectively. The WHO was cited as a partner by regional health officials in Afar and Tigray. In addition, study participants referenced EPHI and the MOH for their technical support.

A few key informants emphasized the difference between trainings – which were longer, content-specific, and detailed versus orientations, which seemed to provide an overview of COVID-19 signs and symptoms, transmission, methods of prevention and that were designed for non-technical staff. Health center directors often raised this distinction, implying that orientations were insufficient for staff preparedness.

Despite their shortcomings, key informants stressed the impact of these trainings on worker attitude and performance. "*Prior to training, health professionals panicked about COVID-19. The training changed their mindsets and they now believe that it is possible to tackle this pandemic.*" (Benishangul-Gumuz, Regional Health Bureau).

COVID-19 supplies and equipment: Health officials and providers across the country noted a shortage of COVID-19 supplies and equipment, particularly personal protective equipment (PPE), at the beginning of the pandemic. For some, access to PPE remained a problem as late as September/October 2020, when the interviews were conducted. Health officials reported that, initially, they faced a shortage of facemasks and protective gowns, and to a lesser extent, gloves.

Access to other supplies and equipment was also a challenge. Health officials reported that they lacked the beds, linens, and medical devices needed to furnish isolation and treatment centers. Hand sanitizers and cleaning materials were also in short supply. A zonal health official in Tigray spoke about their struggle to obtain reagents for laboratories. And health leaders in Somali and Harari regions raised concerns about their ability to deploy and transport RRTs with few cars. Problems accessing non-PPE materials, however, were not uniform: materials that were scarce in one region were available in others. For example, a zonal health official in Addis Ababa stated "*There were no problems with sanitizers.*" While, according to a health center director in Harari, "*There was a greater shortage of sanitizers than gloves.*"

In the early months of the pandemic, the challenge in purchasing COVID-19 supplies and equipment, particularly PPE, was largely due to supply and demand mismatch. The demand for face masks, gloves, and sanitizers outpaced their production. Even when health centers or offices had the budget to purchase PPE, the items were not available in the market. *"At the beginning it was challenging, there was almost no PPE. There was no production of PPE and supply. Even though we tried to buy from the market, it was not available. Later when we invested in PPE - sanitizers, masks and other items - it was challenging financially. It affected the other services significantly."* (Tigray, Health Facility 1).

Health officials used different strategies to procure PPE and other needed materials. Early on, they used what they had in stock. Some repurposed materials that had been in storage for public health

emergencies such as Ebola and cholera. Health centers in particular used a combination of their own revenues and through assistance from regional health bureaus or city administrations to get needed supplies. However, regional health bureaus, zonal health departments, and woreda health offices – relied on budget allocations by administrative bodies and private donations. There was also a shift of budget from other services to COVID. A regional health bureau official from Addis Ababa explained his bureau shifted their budget and "*used finance allocated for non-COVID services to buy PPEs and other resources, which ultimately affected the essential health services*…."

The private sector played a role in meeting demands for COVID-19 supplies and equipment. Businesses, non-governmental organizations, and individuals, both in Ethiopia and abroad, were mobilized to support the health system in this effort. A zonal health leader in Amhara indicated that international non-governmental organizations (NGOs) were instrumental in procuring supplies – like alcohol and sanitizers – that were difficult for health authorities to purchase from the market. Yet, while NGOs and individuals made donations, businesses in a few regions - ranging from microenterprises in Tigray to an industrial park in Sidama - manufactured needed supplies.

In a few regions - Harari, Addis Ababa, Afar and Amhara – study participants across different tiers of the health system reported that their supply issues had been resolved after the initial run on the market. Most received support from a city administration, and health centers in Afar and Addis Ababa supplemented this support by using internal revenues.

Despite their efforts to obtain needed materials, most study participants shared that the PPE items available were inadequate either in quality or, more often, in number. To conserve PPE, health officials resorted to rationing or recycling. At a health center in Addis Ababa, each health professional received 50 masks that were expected to last for 90 days. Health center officials in Oromia requested that health workers wash and reuse masks that were intended to be worn for only four hours. A regional

health bureau official from Amhara spoke about prioritizing PPE for professionals who worked in laboratories and isolation and treatment centers "*even though all health workers were demanding all PPE...*" In the end, an inadequate supply of PPE made it difficult to follow guidelines meant to protect health care providers and clients, which in turn, heightened fear of infection from all sides.

3. Risk Communication and Community Engagement

Risk communication and community engagement was central to the response effort from the beginning of the pandemic. At regional and zonal levels, risk communication teams in incident management offices developed and disseminated messages with support from partner organizations and local government communications offices. Printed materials, radio, and television were commonly used to transmit information, with higher-level offices relying on broadcast media. Regional and zonal offices ran ads on local radio and television stations about the pandemic. Experts appeared on live television or radio programs to answer audience questions or present the latest information about the virus. As the following quote attests, collaborations with international NGOs, academic institutions, and professional associations helped regional and zonal leaders to maximize their communication efforts: "*We also worked with the Amhara Physicians' Association, clinicians, and public health experts who made live appearances on television programs in collaboration with Bahir Dar University*." (Amhara, Regional Health Bureau).

To a lesser extent, regional and zonal health offices also used new media, particularly Facebook, to share information and updates. The regional health bureau in Tigray, for instance, posted the daily number of confirmed cases in the region on its website and Facebook page. Visitors to these sites could also find information about COVID and steps local authorities were taking to contain the pandemic. In addition to traditional and new media, health officials in Sidama and SNNP collaborated with artists to develop music and drama about COVID: "*Artists in the Sidama region communicated*

messages through songs and drama, for adults and children, on how to prevent the disease." (Sidama, Regional Health Bureau). A woreda health official from SNNP spoke of a "holistic" song produced by senior Wolaita musicians that gained popularity with rural communities. In Oromia, community and religious leaders played a role in community education. *"We provided information to the community through religious leaders, cultural leaders - Aba Geda, and different media. People in our region have received sufficient information about prevention and have used that information to keep themselves safe."* (Oromia, Regional Health Bureau).

Woreda health offices and health centers commonly relied on printed materials (leaflets, brochures, posters, and banners) to spread information about the coronavirus. Regional or zonal offices created these materials in local languages and sent them to the lower tiers for distribution. Health extension workers (HEWs), volunteers, and care providers handed out brochures and fliers in the community and during house-to-house visits. Posters and banners were displayed in crowded markets and busy streets.

Intensive outreach took place at different sites - homes, work places, places of worship, and health facilities. Health professionals, teachers, and volunteers visited households in Tigray and in Somali regions and also health extension workers, traditional birth attendants, and family health teams visited households. Health professionals also used loud speakers, often driving through city streets or villages, to maximize their reach. Health centers and woreda health offices made a deliberate effort to target vulnerable communities. For example, a health center in Amhara conducted outreach to the homeless and a woreda in Addis Ababa shaped its message about preventive measures to address the concerns of community members with chronic illnesses.

Risk communication messages stressed the importance of maintaining hand hygiene (using soap and water or hand sanitizers), wearing masks, keeping a physical distance. Messages also covered the signs and symptoms of the disease and provided hotline numbers to call for additional information or to

report suspected cases. During household visits, health extension workers demonstrated proper hand washing, physical distancing, and mask wearing techniques. *"HEWs did house-to-house visits in their assigned areas or kebeles. They supplied households with soaps and businesses with hand sanitizers. They taught households how to prevent COVID-19, advised them to stay home, and to wear masks if they were in public. They also gave out leaflets with the emergency telephone number*." (Somali, Health Facility 2).

A health center in Harari encouraged community members with chronic conditions to continue taking their medications. Still, financial, political, and environmental pressures placed limits on risk communication efforts in some regions. A representative from the Addis Ababa regional health bureau said "*the rate of mass media utilization is not as expected due to the expense*." Also, in Sidama, a woreda health official noted that "*performance was very strong initially, but it has become weak recently because of cost and our lack of capacity*" in reference to the use of mobile public announcement systems to disseminate information about the virus. Outreach was stymied in rural parts of Harari due to security issues and heavy rainfall that made roads inaccessible.

4. Surveillance, Rapid Response Teams, and Case Investigation

Health officials relied on syndromic screening to isolate possible cases of COVID-19. Screenings were based on a case definition published by the Federal Ministry of Health. The most recent definition was published in May 2020 and a health center director in Addis Ababa suggested that even prior to the most recent change, modifications were being made to the definition at lower levels (38). Study participants also highlighted the limits to pointed syndromic-based case definitions in the context of high rates of asymptomatic infections.

Health extension workers conducted house-to-house screenings in three rounds with a testing campaign that occurred in August 2020. Community screenings targeted high-risk groups and places where transmissions were most likely to occur: long-distance bus drivers for instance, were prioritized

for screening in Amhara and Tigray and screenings took place at bus station and police stations in Gambella. Community members were also encouraged to contact health authorities if anyone in the community showed signs or symptoms of the disease.

Health centers set up triage areas to screen every patient who entered their compounds. Screeners used a checklist to identify suspected cases. The initial screening consisted of a temperature check and questions probing signs and symptoms of acute respiratory infection. Patients with fever and cough were escorted to an isolation room in the health center where they were examined further by a clinician. Patients who were suspected of coronavirus remained in the isolation room until they were tested.

Many health centers set up isolation areas by converting a room or unused area in their facilities. In some centers, the conversion of spaces encroached on existing services, as in the case of health centers in Benishangul-Gumuz where waiting rooms for expectant mothers were transformed into isolation rooms for suspected coronavirus cases. Isolation rooms were outfitted with beds, oxygen cylinders, and pulse oximeters in Addis Ababa and Tigray.

Following a suspicious finding in the community, a RRT was called to conduct an investigation. The team - which typically consisted of a clinician, an epidemiologist, an environmental health expert, a health educator, and a lab technician – then transported individuals suspected of coronavirus to a quarantine center where they were tested and remained until their results were known.

Depending on the region, either staff from the EPHI or members of RRTs were responsible for sample collection. In August, the federal government undertook a nation-wide campaign to gauge the level of infection in the country and measure community understanding of COVID prevention measures. Vulnerable groups, such as those with chronic conditions, were specifically targeted for testing during the campaign.

The RRTs were also responsible for transporting samples to the nearest laboratory. Each region had at least one laboratory and many were located on the campuses of major universities. Before the regional laboratories were set up, tests were sent to laboratories in Addis Ababa or outside the country. Key informants in Oromia and Tigray noted that laboratories struggled to report test results in a timely manner. Delays were reportedly due to a lack of coordination, limited diagnostic machines, and a lack of reagents. *"Quarantine is located at another site…. but the problem is test results are not reported timely and a lot of complains are coming from people staying at the quarantine center. Results are being reported 9 or 10 days after they are expected, which is extremely frustrating. …"* (Tigray, Woreda Health Office).

5. Case Management and infection prevention and control

Case management: Individuals in quarantine were released after seven days if they tested negative. Otherwise, they were transferred to an isolation or treatment center. Rapid response teams conducted contact tracing and suspected contacts were placed in quarantine and tested. Health authorities established quarantine and isolation centers in schools and universities (which were closed at the time (2020) after the state of emergency was declared) and hotels donated by local business owners.

Health facilities, usually hospitals, terminated routine services to serve as treatment centers. Zonal health officials in Sidama opted to use space donated by local businesses for COVID-19 treatment rather than interrupt the delivery of essential services at health facilities. As the number of cases that required hospitalization grew, health authorities expanded the number of treatment-centers in each region – often selecting schools and universities as supplementary sites. A regional health bureau official in Afar reported that, "*with the surge of infections, severe cases of COVID-19 were placed in hospitals and milder cases were treated in non-traditional care facilities*". The policy changed to allow mild and

asymptomatic cases to isolate at home as the state of emergency came to an end and schools prepared to re-open. Rapid response teams determined who could isolate at home and regularly followed-up.

Infection Prevention and Control: Health centers undertook precautions to ensure the safety of their workers and patients. They set up washing stations where patients were required to wash their hands before proceeding to their appointments. They limited entry only to patients, placed markers two meters apart so that patients could adhere to physical distancing requirements, and disinfected essential departments. Some centers had two gates, one for entry and another for exit. *"In addition, guards were given additional work to make sure that every client entered to our health center washed their hands using water and soap available at the gate"*. (Oromia, Facility 2).

In regions across Ethiopia, community-based infection prevention and control was largely the responsibility of regional, zonal, and woreda health offices. Many installed handwashing facilities in crowded areas. A woreda in SNNP temporarily housed "street dwellers" who were considered high-risk for infection and provided them with food and hygiene materials. A zonal health department in Sidama disinfected sidewalks and office buildings every 15 days. Higher-level offices collaborated with the police to enforce or promote infection control measures in markets, restaurants, and passenger cars. They also organized volunteers, particularly youth and members of the women's development army (volunteer community health workers), to educate and encourage people to adhere to prevention strategies.

Discussion

Similar to many other African countries, different regions of Ethiopia acted early by implementing screening measures at airports and border crossings before the first case of coronavirus was detected (39). Following the confirmation of the first case, sub-national government structures from regional councils to kebeles set up multisectoral coordinating committees that were responsible for planning,

organizing, and monitoring local responses. Research indicates that local governments also played a vital role in the pandemic response in other low- and middle-income countries. Similar to our findings, studies from China and India showed that local government efforts were multisectoral and focused on resource mobilization, infection control, and risk communication (40-42). A county government outside Wuhan, China, for example, organized “functional groups” that, among other responsibilities, procured personal protective equipment from different sources for local public hospitals and community volunteers (41). These studies also found out that local governments provided aid to needy individuals and households, enforced disease control measures such as physical distancing and screening and quarantine of travellers, and created public awareness (40,41).

These examples, as well as our own experience in Ethiopia, showed the potential for local governments to play a critical role in public health emergencies and other disasters. As Dutta and Fisher have argued, decentralized government institutions are better positioned to carry out the basic functions of disease control because they have a deeper understanding of community needs, can respond more efficiently to crises, and hold greater legitimacy with constituents than distant government officials (40). Unfortunately, our study showed that the role of local governments was not sustained over the course of the pandemic – a finding that was not reflected in other studies.

Initially, the sub-national response in Ethiopia also embodied a whole-of-society approach that was promoted and reflected in the literature. This approach endorses collaboration across sectors based on the notion that no single body or institution can capably respond to complex emergencies or social problems (43). Again, studies from India and China found that local governments mobilized community members to volunteer in the response effort (40,44,45). Also, a survey of medical students, health workers, and community service workers in Vietnam showed strong support for involving youth groups, women’s associations, religious leaders, and local organizations in community education,

surveillance, and contact tracing during the pandemic which is similar to our study particularly at the initial phase (46). In our study, participants highlighted and praised civic society institutions, volunteers, and businesses for engaging in a variety of activities, ranging from community education and the provision of supplies and facilities to the enforcement of infection control measures. This was a very good lesson. But, six months after the start of the pandemic, the study participants observed a decline in multisectoral engagement and warned of the health sector's capacity to control the pandemic in isolation.

Because of underinvestment in health care, many low-income countries suffer from a shortage of critical health inputs including, supplies, infrastructure, and health professionals. To fill the gap in human resources, for instance, experts advocate the use of community health workers in both maintaining essential health services and containing the pandemic (47,48). This is viewed as a feasible solution in settings where community health workers outnumber clinicians and function as trusted sources of health information and services (49).

Our study showed that community health workers (known as health extension workers or professionals) in Ethiopia were key to COVID response strategies. They engaged in community education - principally through house-to-house visits - performed screenings, and assisted RRTs with contact tracing. A rapid evidence synthesis found that community health workers in low- and middle-income countries played similar roles in the pandemic response (50). Furthermore, when essential health resources showed signs of decline in Ethiopia, community health workers were tasked with alleviating community members' fears of accessing health services and re-engaging patients who were lost to follow-up. In many areas, responsibilities related to COVID-19 were added to their existing workload, creating low morale and push-back from health extension workers as the time progressed. The rapid evidence synthesis showed that improving benefits, providing training, and recognizing the

contributions of community health workers in high-profile events could bolster their recruitment and retention (50).

In addition to mobilizing health extension workers, sub-national health systems in Ethiopia addressed the surge in the health workforce by reassigning health professionals, cancelling leaves of absence, recruiting new health workers, and bringing retired or private sector professionals into the workforce. While comparable research in low-income countries was not available, a study of 44 countries in Europe and North America showed that the governments of wealthy nations used mix of policy options similar to what we found out (51). The most common strategies involved maintaining the existing workforce by extending work hours, moving staff from part-time to full-time, or suspending annual leaves. These governments also recruited medical and nursing students (a strategy that was not mentioned by participants in our study), redeployed workers to facilities or areas with the greatest need, and enlisted retired, foreign, and private sector professionals. Most countries used at least two strategies. While the impact of these measures in Europe and North America has not been studied, our study participants linked a weakening of the essential health services to the redeployment of staff to the COVID-19 response.

An important factor in containing COVID-19 was protecting the health workforce from infection. Study participants shared their struggles to acquire PPE, particularly face masks and gowns, at the beginning of the pandemic. Due to constrains in production, PPE was limited and costly. Ethiopia was not unique in this regard; countries worldwide faced a shortage of PPE and price hikes at the start of the pandemic (52). But low-income countries were disproportionately affected because of their relatively weak bargaining positions in the global supply chain, low capacity in production of supplies, limited purchasing power, and low hands-on reserves of PPE (53-55).

When production began to accelerate, study participants accessed key protective gear through different means, including the engagement of local businesses. Local production was a solution to the shortage of PPE in a number of low-income countries: the governments of Uganda, Tanzania, Kenya, Nigeria, Liberia, and Ghana, for instance, directed local manufacturers toward the production of PPE (53-55). At a more grassroots level, a government hospital in Dar es Salaam, Tanzania collaborated with seamstresses, business owners, community members, and NGOs to produce reusable face masks, gowns, scrubs and caps for its staff (54). Despite their efforts to meet the demand in PPE, most participants in our study were unsatisfied with the quantity of PPE at their disposal. Our finding is corroborated by a survey of health care workers at a public hospital in Addis Ababa in which over three-fourth of the respondents reported an inadequate supply of PPE at their hospital (56).

Risk communication and community engagement was central to the response effort in Ethiopia. Regional and zonal offices used broadcast media, and to a lesser extent new media such as Facebook, to disseminate information about the coronavirus. With support from international NGOs, academic institutions, and professional associations, they ran ads on local television and radio stations and invited experts to speak about the pandemic on broadcast media. However, risk communication efforts were limited in some areas by financial, political, and environmental obstacles, Most COVID-related studies conducted in Ethiopia examined knowledge, attitudes, and practices among community members, showing generally high rates of knowledge and poor prevention practices.

Limitations of the study: Due to national and state level lockdowns the investigators could not go out to the field thus limiting the potential for extra lens to note field observations not captured by the interviews. However, the authors have tried to overcome this by assigning a supervisor for each interviewer in each region. We noted that data collected from 2 of the regions was not good enough in terms of depth and probes affecting the richness in some of the topics. This might be due to lack of

experience of those interviewers in applying the different techniques of qualitative methods. However, the impact was minimal as the sample was large enough to explore the topics of interest.

Conclusion:

Sub-national health structures waged a swift and coordinated response to COVID-19 at the start of the pandemic. They drew on all available resources - from their own expertise and workforce to local governments and businesses, academic institutions, and community members to mitigate the spread of the virus. However, the response was strained from the beginning – due to a shortage of health workers, space, equipment and supplies – and began to fray as the pandemic worsened. The multisectoral response that started with strong political commitment and drove the initial response declined within six months of the pandemic. As the number of infections in Ethiopia rise, we recommend: reengaging local administrations and actors outside the health sector, including academic institutions and local businesses, in the response effort; strengthening resource mobilization for COVID supplies and equipment; improving testing capacity across the country; and collaborating with local media and community leaders to strengthen community adherence to prevention measures. There is a need to invest in the basics of the health system – health workers, supplies and equipment, and infrastructure – as well as specific interventions to shore up the COVID response and restore the multi-faceted response. The study underlines engagement of local governments is key to take immediate action and sustain the local response against COVID-19.

Authors' contributions

GM – contributed in conceptualization, proposal writing, formal analysis and writing original draft, review and editing; EW – contributed in conceptualization, methods, proposal writing, review and editing: FN – contributed in conceptualization, proposal writing, review, and editing: TN - contributed

in data curation, formal analysis and writing original draft; MT - contributed in proposal writing, review and editing: AD – contributed in proposal writing, review and editing.

Acknowledgements

We would like to acknowledge the study participants and their respective regional health bureaus, zonal health departments, woreda health offices, and health centers for agreeing to take part in this study. We are also grateful to the data collectors and supervisors, staff at Ethiopian Public Health Institute and the International Institute for Primary Health Care-Ethiopia for their technical support.

Funding

This study was funded by the Bill and Melinda Gates Foundation through the International Institute for Primary Health Care - Ethiopia.

Disclaimer: The contents of this report are solely the responsibility of the authors and do not necessarily represent the official views of the Bill and Melinda Gates Foundation

Availability of data and materials

The datasets used and/or analysed during the current study are available from the corresponding author on reasonable request

Ethics approval and consent to participants

Approved by the Ethiopian Public Health Institute – Institutional Review Board (EPHI IRB # 280-2020). The IRB approved the informed verbal consent to minimise contact through paper/pen exchange considering the COVID-19 transmission.

Consent for publication

Not required

Competing interests

The authors declare that there are no competing interests

Authors details

Getnet Mitike* (MD, PhD)[1], Frehiwot Nigatu (MD, MPH)[1], Eskinder Wolka (PhD),[1]Atkure Defar (PhD)[2], Masresha Tessema (PhD)[2], TezitaNigussie (MSW, MPH)[1]

[1]International Institute for Primary Health Care-Ethiopia; [2] Ethiopian Public Health Institute

*=corresponding author (email: getnetmk@gmail.com)

References

1. World Health Organization. WHO Director General's Opening Remarks at the media briefing on COVID-19, 11 March 2020. https://www.who.int/director-general/speeches/detail/who-director-general-s-opening-remarks-at-the-media-briefing-on-covid-19---11-march-2020
2. World Health Organization. *WHO Coronavirus (COVID-19) Dashboard*. Available from: https://covid19.who.int/.
3. National Threat Initiative, Johns Hopkins Bloomberg School of Public Health Center for Health Security. *Global health security index: building collective action and accountability*. NTI. 2019. Available at: https://www.ghsindex.org/wp-content/uploads/2019/10/2019-Global-Health-Security-Index.pdf.
4. Kandel N, Chungong S, Omaar A, Xing J. Health security capacities in the context of COVID-19 outbreak: an analysis of International Health Regulations annual report data from 182 countries. *The Lancet*. 2020;395(10229):1047-53. https://doi.org/10.1016/S0140-6736(20)30553-5.
5. Lucero-Prisno DE, Adebisi YA, Lin X. Current efforts and challenges facing responses to 2019-nCoV in Africa. *Global Health Research Policy*. 2020;5,21. https://doi.org/10.1186/s41256-020-00148-1.
6. El-Sadr WM, Justman J. Africa in the path of COVID. *New England Journal of Medicine*. 2020;383: e11. doi:10.1056/NEJMp2008193.
7. Nyenswah T. *Africa has a COVID-19 timebomb to defuse*. Available from: https://www.weforum.org/agenda/2020/04/africa-covid-19-time-bomb-defuse/.
8. Elston JWT, Cartwright C, Ndumbi P, Wright J. The health impact of the 2014–15 Ebola outbreaks. *The Royal Society of Public Health*. 2016; 143:60-70. https://doi.org/10.1016/j.puhe.2016.10.020.
9. Partnership for Evidence-based Response to COVID-19. *Finding the balance: public health and social measures in Ethiopia*. PERC. 2020. Available at: https://preventepidemics.org/wp-content/uploads/2020/09/09082020-ethiopia.pdf.
10. Mohammed H, Oljira L, Roba KT, Yimer G, Fekadu A, Manyazewal T. Containment of COVID-19 in Ethiopia and implications for tuberculosis care and research. *Infectious Diseases of Poverty*. 2020;9(1):131. https://doi.org/10.1186/s40249-020-00753-9.
11. Kebede A, Lanyero B, Beyene B, Mandailia ML, Melese D, Girmachew F, et al. Expanding molecular diagnostic capacity for COVID-19 in Ethiopia: operational implications, challenges and lessons learnt. *Pan African Medical Journal*. 2021; 38:68. *https://doi.org/10.11604/pamj.2021.38.68.27501*.
12. Adesanya OA. Government preparedness and response towards COVID-19 outbreak in Nigeria: A retrospective analysis of the last 6 months. *Journal of Global Health*. 2020;10(2). https://doi.org/10.7189/jogh.10.020382.
13. Etteh CC, Adoga MP, Ogbaga CC. (2020). COVID-19 response in Nigeria: Health system preparedness and lessons for future epidemics in Africa. *Ethics, Medicine, and Public Health*. 2020;15:100580. https://doi.org/10.1016/j.jemep.2020.100580.
14. Staunton C, Swanepoel C, Labuschaigne M. Between a rock and a hard place: COVID-19 and South Africa's response. *Journal of Law and Biosciences*. 2020;7(1). https://doi.org/10.1093/jlb/lsaa052.
15. Umviligihozo G, Mupfumi L, Sonela N, Naicker D, Obuku EA, Koofhethile C, et al. Sub-Saharan Africa preparedness and response to the COVID-19 pandemic: a perspective of early career African scientists. *Wellcome Open Research*. 2020;10(5):163. https://doi.org/10.12688/wellcomeopenres.16070.3.
16. Lumu I. COVID-19 response in Sub-Saharan Africa: lessons From Uganda. *Disaster Medicine and Public Health Preparedness*. 2020;14(3): e46-e48. https://doi.org/10.1017/dmp.2020.248.
17. Elhakim M, Tourab SB, Zouiten A. COVID-19 pandemic in Djibouti: epidemiology and the response strategy followed to contain the virus during the first two months, 17 March to 16 May 2020. *PLoS One*. 2020;15(12):e0243698. https://doi.org/10.1371/journal.pone.0243698.
18. Musa TH, El Bingawi HM, Musa IH, Mohammed LA, Arbab MA, Musa HH. COVID-19 in Sudan: Response towards control and prevention. *Medical Journal of Malaysia*. 2020;75(4):403-405. http://www.e-mjm.org/2020/v75n4/COVID-19-in-Sudan.pdf.

19. Aluga MA. Coronavirus Disease 2019 (COVID-19) in Kenya: Preparedness, response and transmissibility. *Journal of Microbiology, Immunology and Infection*. 2020;53(5):671-673. https://doi.org/10.1016/j.jmii.2020.04.011.
20. Tchole AIM, Li ZW, Wei JT, Ye RZ, Wang WJ, Du WY, et al. Epidemic and control of COVID-19 in Niger: quantitative analyses in a least developed country. *Journal of Global Health*. 2020;10(2):020513. https://doi.org/10.7189/jogh.10.020513.
21. Murewanhema G, Burukai T, Mazingi D, Maunganidze F, Mufunda J, Munodawafa D, et al. A descriptive study of the trends of COVID-19 in Zimbabwe from March-June 2020: policy and strategy implications. *Pan African Medical Journal*. 2020;37(Suppl 1):33. https//doi.org/ 10.11604/pamj.supp.2020.37.33.25835
22. Kebede Y, Yitayih Y, Birhanu Z, Mekonen S, Ambelu A. Knowledge, perceptions and preventive practices towards COVID-19 early in the outbreak among Jimma university medical center visitors, Southwest Ethiopia. *PLoS One*. 2020;15(5): e0233744. https://doi.org/10.1371/journal.pone.0233744.
23. Kassie BA, Adane A, Tilahun YT, Kassahun EA, Ayele AS, Belew AK. Knowledge and attitude towards COVID-19 and associated factors among health care providers in northwest Ethiopia. *PLoS One*. 2020;15(8):e0238415. http://doi.org/10.1371/journal.pone.0238415.
24. Aweke Z, Jemal B, Mola S, Hussen R. Knowledge of COVID-19 and its prevention among residents of the Gedeo zone, south Ethiopia: sources of information as a factor. *Current Medical Research and Opinion*. 2020;36(12):1955-1960. https://doi.org/10.1080/03007995.2020.1835854.
25. Tadesse DB, Gebrewahd GT, Demoz GT. Knowledge, attitude, practice and psychological response toward COVID-19 among nurses during the COVID-19 outbreak in northern Ethiopia, 2020. *New Microbes and New Infections*. 2020;38:100787. https://doi.org/10.1016/j.nmni.2020.100787
26. Azene ZN, Merid MW, Muluneh AG, Geberu DM, Kassa GM, Yenit MK, et al. Adherence towards COVID-19 mitigation measures and its associated factors among Gondar city residents: a community-based cross-sectional study in northwest Ethiopia. *PLoS One*. 2020;15(12):e0244265. https://doi.org/10.1371/journal.pone.0244265.
27. Kassa AM, Mekonen AM, Yesuf KA, Woday Tadesse A, Bogale GG. Knowledge level and factors influencing prevention of COVID-19 pandemic among residents of Dessie and Kombolcha City administrations, north-east Ethiopia: a population-based cross-sectional study. *British Medical Journal Open*. 2020;10(11): e044202. https://doi.org/10.1136/bmjopen-2020-044202.
28. Asemahagn MA. Factors determining the knowledge and prevention practice of healthcare workers towards COVID-19 in Amhara region, Ethiopia: a cross-sectional survey. *Tropical Medicine and Health*. 2020; 48:72. https://doi.org/10.1186/s41182-020-00254-3.
29. Girma S, Alenko A, Agenagnew L. Knowledge and precautionary behavioral practice toward COVID-19 among health professionals working in public university hospitals in Ethiopia: A web-based wurvey. *Risk Management Healthcare Policy*. 2020 Aug 24; 13:1327-1334. https://doi.org/10.2147/RMHP.S267261.
30. Desalegn Z, Deyessa N, Teka B, Shiferaw W, Hailemariam D, Addissie A, et al. COVID-19 and the public response: Knowledge, attitude and practice of the public in mitigating the pandemic in Addis Ababa, Ethiopia. *PLoS One*. 2021 Jan 7;16(1):e0244780. https://doi.org/10.1371/journal.pone.0244780.
31. Bekele D, Tolossa T, Tsegaye R, Teshome W. The knowledge and practice towards COVID-19 pandemic prevention among residents of Ethiopia. An online cross-sectional study. *PLoS One*. 2021;16(1):e0234585. Available from: https://doi.org/10.1371/journal.pone.0234585.
32. Amsalu B, Guta A, Seyoum Z, Kassie N, Sema A, Dejene W, et al. Practice of COVID-19 prevention measures and associated factors among residents of Dire Dawa city, eastern Ethiopia: community-based study. *Journal of Multidisciplinary Healthcare*. 2021; 14:219-228. https://doi.org/10.2147/JMDH.S292409.
33. Yoseph A, Tamiso A, Ejeso A. Knowledge, attitudes, and practices related to COVID-19 pandemic among adult population in Sidama regional state, southern Ethiopia: a community based cross-sectional study. *PLoS One*. 2021;16(1):e0246283. https://doi.org/10.1371/journal.pone.0246283.
34. Desalegn Z, Deyessa N, Teka B, Shiferaw W, Yohannes M, Hailemariam D, et al. Evaluation of COVID-19 related knowledge and preparedness in health professionals at selected health facilities in a resource-limited

setting in Addis Ababa, Ethiopia. *PLoS One*. 2021;16(2): e0244050. https://doi.org/10.1371/journal.pone.0244050..
35. Adhena G, Hidru HD. Knowledge, Attitude, and Practice of High-Risk Age Groups to Coronavirus Disease-19 Prevention and Control in Korem District, Tigray, Ethiopia: Cross-Sectional Study. *Infection and Drug Resistance*. 2020 Oct 23;13:3801-3809. https://doi.org/10.2147/IDR.S275168.
36. World Health Organization. *Primary health care systems (PRIMASYS): case study from Ethiopia*. WHO. 2017. Available at: https://www.who.int/alliance-hpsr/projects/alliancehpsr_ethiopiaprimasys.pdf?ua=1.
37. Ulin PR, Robinson ET, Tolley EE. *Qualitative Methods in Public Health: A Field Guide for Applied Research*. San Francisco: Jossey-Bass; 2005.
38. Ethiopian Public Health Institute. *COVID-19 suspected case definition*. Available at: https://www.ephi.gov.et/images/novel_coronavirus/EPHI_PHEOC_COVID-19_Suspected_Case_Definition_05172020_Eng.pdf.
39. Senghore M, Savi MK, Gnangnon B, Hanage WP, Okeke IN. Leveraging Africa's preparedness towards the next phase of the COVID-19 pandemic. *The Lancet Global Health*. 2020;8(7):E884-E885. https://doi.org/10.1016/S2214-109X(20)30234-5.
40. Dutta A, Fischer HW. The local governance of COVID-19: Disease prevention and social security in rural India. *World Development*. 202;138:105234. Available from: https://doi.org/10.1016/j.worlddev.2020.105234.
41. Han Z, Ai X, Veuthey J. How is COVID-19 Being Contained in China? An Observational Study on the Local Level. *Disaster Medicine and Public Health Preparedness*. 2020:1-8. https://doi.org/10.1017/dmp.2020.49.
42. Sadanandan R. Kerala's response to COVID-19. *Indian Journal of Public Health*. 2020;64(6):99-101. https://www.ijph.in/text.asp?2020/64/6/99/285594.
43. Dubb SS. Coronavirus pandemic: applying a whole-of-society model for the whole-of-the world. *The British Journal of Oral & Maxillofacial Surgery*. 2020;*58*(7):838–842. https://doi.org/10.1016/j.bjoms.2020.05.009.
44. Miao Q, Schwarz S, Schwarz G. Responding to COVID-19: community volunteerism and coproduction in China. *World Development*. 202;137:105128. https://doi.org/10.1016/j.worlddev.2020.105128.
45. Li CH, Tan CX, Wu AH, Gao CQ. COVID-19: the role of community in China's response. *Journal of the Royal Society of Medicine*. 2020;113(7):280-281. https://doi.org/10.1177/0141076820935344.
46. Le HT, Mai HT, Pham HQ, Nguyen CT, Vu GT, Phung DT, et al. Feasibility of intersectoral collaboration in epidemic preparedness and response at grassroots levels in the threat of COVID-19 pandemic in Vietnam. *Frontiers in Public Health*. 2020; 8:589437. https://doi.org/10.3389/fpubh.2020.589437.
47. Boyce MR, Katz R. Community health workers and pandemic preparedness: current and prospective roles. *Frontiers in Public Health*. 2019; 7:62. https://doi.org/10.3389/fpubh.2019.00062.
48. Palafox B, Renedo A, Lasco G, Palileo-Villanueva L, Balabanova D, McKee M. Maintaining population health in low- and middle-income countries during the COVID-19 pandemic: Why we should be investing in community health workers. *Tropical Medicine and International Health*. 2021:26: 20-22. https://doi.org/10.1111/tmi.13498.
49. Nepomnyashchiy L, Dahn B, Saykpah R, Raghavan M. COVID-19: Africa needs unprecedented attention to strengthen community health systems. *The Lancet*. 2020;396(10245):150-152. https://doi.org/10.1016/S0140-6736(20)31532-4.
50. Bhaumik S, Moola S, Tyagi J, Nambiar D, Kakoti M. Community health workers for pandemic response: a rapid evidence synthesis. *BMJ Global Health*. 2020;5(6):e002769. http://dx.doi.org/10.1136/bmjgh-2020-002769.
51. Williams GA, Maier CB, Scarpetti G, Giulio de Belvis A, Fattore G, Morsella A, et al. What strategies are countries using to expand health workforce surge capacity during the COVID-19 pandemic? *Eurohealth*. 2020;26(2):51-57. https://apps.who.int/iris/handle/10665/336296.
52. Burki T. Global shortage of personal protective equipment. *The Lancet: Infectious Diseases*. 2020;20(7):785-86. https://doi.org/10.1016/S1473-3099(20)30501-6.
53. McMahon DE, Peters GA, Ivers LC, Freeman EE. Global resource shortages during COVID-19: Bad news for low-income countries. *PLoS Neglected Tropical Diseases*. 2020;14(7): e0008412. https://doi.org/10.1371/journal.pntd.0008412.

54. Pieterse P, Dickson C, Ndyetabula L, Hardeman M, Scanlan P. Locally produced personal protective equipment can offer hospital staff protection against Covid-19 if combined with surgical masks and rigorous personal protective equipment cleaning routine. *The International Journal of Health Planning and Management*. 2021;36(2):587–589. https://doi.org/10.1002/hpm.3080.
55. Gage A, Bauhoff S. *Health systems in low-income countries will struggle to protect health workers from COVID-19*. Center for Global Development. 2020. Available at: https://www.cgdev.org/blog/health-systems-low-income-countries-will-struggle-protect-health-workers-covid-19.
56. Deressa W, Worku A, Abebe W, Gizaw M, Amogne W. Availability of personal protective equipment and satisfaction of healthcare professionals during COVID-19 pandemic in Ethiopia. *medRxiv*. 2020: 20223149. https://doi.org/10.1101/2020.10.30.20223149.

Click here to access/download
Supporting Information
Guides_all.pdf
