# Supplementary material for: Health system response to COVID-19 among primary health care units in Ethiopia: A qualitative study
Source: PLoS One. 2023 Feb 10;18(2):e0281628. doi: 10.1371/journal.pone.0281628 (PMC9916627; doi:10.1371/journal.pone.0281628)
Supplement: Attachment — Submitted filename: Response to reviewers .docx [file pone.0281628.s004.docx]

Response to reviewers’ comments (Reviewer 1 and Reviewer 2)

*Page# and Line # refers to the main proposal – from abstract to references

| ***Section, page, paragraph** | **Reviewer 1 comment** | **Response** |
| --- | --- | --- |
| General | Editorial comments | Thank you for your valuable comments and editorial inputs. Almost all of them are accepted. |
| **Abstract**  Line 25 | What is the meaning of clinicians? | Changed to healthcare providers (this is to avoid confusion). Also it’s elaborated. This would include those who provide treatment and supportive care… Drs and Nurses |
| Line 26 | Elaborate the meaning of treatment centers. | Accommodated: where care was provided for patients with COVID-19 |
| Line 31-33 | The care for mild cases was shifted to home-based isolation as the number of cases increased and spaces became limited. | Editorial: Accommodated |
| **Introduction:** paragraph 1  Line 55-57 | Editorial | Accepted |
| Paragraph 2 and 3 | Editorial | Accepted |
| **Materials and Methods:**  Selection of participants  Line 86 | You said you study ten regions and two cities then take from each 5 persons so the number should be 60 participants. | This is correct. However the # of respondents is 59. This is explained on the result’s section, paragraph 1  Line 138-139 |
| **Results:**  Themes and subthemes  Line153,154,198,199  224,238,283  342, 393, 394, 400 | Numbering/highlighting subtitles | Accommodated (highlighted - bold) |
| page 9 last paragraph  Line 216 | What about professionals and health workers who got infected, how you manage? What the effect of that on the health service? | This was not included in our probing questions and hence not mentioned by respondents. However there were responses regarding access to PPE and other self-protection measures. This was also the time when anyone suspected would be quarantined, isolated and get treated at treatment centers – transition to quarantine and isolation at home was not widely practiced |
| page 14  line 322 | Elaborate what do you mean by chronic illness as they are at high risk for infection. | Elaborated and edited (chronic illness) changed to NCDs which is more commonly used now adays |
| All editorial comments | Editorial comments and deletions | Accepted and # of pages reduced from 30 to 28 |
| **Discussion:**  page 19  Line 435-437 | While the impact of these measures in Europe and North America has not been studied, our study participants linked a weakening of the essential health services to the redeployment of staff to the COVID-19 response. Why? | Explanation included: This might be due to inadequate strategies and financial constraints in recruitment of new staff and redeployment of available health workers in Ethiopia. |
| page 22 last paragraph | Why you do not involve assessment of modalities of online care that need extend rapid access to cellphones and internet for health professionals and users. Also, remote consultations with video, since it facilitates monitoring vital signs and decreased the risk of infection. | They have used it to some extent for instance in training, sharing guidelines (page 10, line 234) and other related information from the MOH. It is also mentioned how Facebook was used (page 13, line 297,298). However, internet connection is not reliable and accessibility is limited at district level where facilities are located far from urban areas (response not included in the text). In contrast it was stated that use of local media and printed materials was common (page 13, line 287, 295 last paragraph and 14, line 301-308 and 309-320) |

|  | **Reviewer 2 Comments** | **Response** |
| --- | --- | --- |
| **Section, page, paragraph and Line #** |  | Thank you for the insightful comments and editorial inputs. Almost all of them are accommodated. |
| **Abstract:**  Page 1, Line 20-21 | From where? Multi sectoral planning and monitoring committee | This is elaborated: at regional, zonal and district levels |
| Line 24-25 | What type of training? | Type of training elaborated፡ detailed description on page 10 (line 224-235) |
| Line 29 | What do you mean that the quantity of resourceful means to protect staff was short of the needs? Need to revise the English. | Edited: resourceful is deleted and sentence checked |
| Line 30 | How effective is house-to-house campaign | It’s very difficult to answer this question with this study; however, it was useful in raising the awareness of people and educating them how to use PPE (wearing masks, washing hands with soap and water and keeping distance) |
| Line 37-39 | Multisectoral response dropped in what ways …quantity, effectiveness, etc. | Edited: Here we are taking about decline in the engagement of stakeholders. (we do not have data why this is happening) but we have included some description in the discussion |
| **Introduction:** 1^st^ paragraph, line 3  Line 51-52 | Provide figures and values | Comment accepted and added: By the end of 2020 (December 28), there had been a total 4,162, 931 confirmed cases and 81,404 deaths |
| **Method and materials:** study setting,  Page 3  1^st^ paragraph, Line 86 | It will be good if these can be put into a figure with the administrative structure side by side with the health system structure on the same figure. | Figure is included under supporting information |
| Multiple pages | Write COVID into COVID-19 | Checked and corrected in all |
| **Results:** page 6  Line 140 and 144 | The authors did not put any unit on their values. 35 what? 35 eggs, bread, months, etc? | Corrected: years is added |
| page 7  line 155-156 | 'In a few' is subjective. You have to be quantitative. in 15%, 24% etc. | Many experts discourage quantitative results in qualitative studies. More descriptions added empathizing traveler entry points: international airports land border crossing neighboring countries (about two-third). However this is not included in the description. |
| page 7  line 164-169 and 175-176 | Agriculture (Livestock) and Environment were not represented? | Agriculture and environment would be included under the corresponding Regional Disaster Risk Management Council. However, this was not mentioned by respondents. A new reference is added to clarify the type of coordinating committees. |
| page 8  Line 182-183 | Affected in what regards? Infected? Livelihood losses, hunger, lost loved ones???? | This is clarified: people living with daily substance, long distance drivers, police stations… |
| page 7  Line 164-169 | How many coordinating committees exist? What is the regularity of this meeting? Was the meeting schedules different for each administrative levels or it was one centrally coordinated meeting? | I have added a new reference that state the details of the coordinating committees and have added some elaborations. This can be extrapolated from the national structures that are described. However, we did not have details regarding their schedules and meetings |
|  | This connotes March 2020, hence the many questions above. | This is related to the previous responses |
| Page 8,  Line 192 | Were there statutory committees for the health centres before these new committees set specifically for COVID-19? How do this (these) coordinate with COVID-19 related committees? | There are different teams for instance management committee. However, we did not probe into these areas as the focus was on structures set for COVID-19 |
| Page 12  Line 263 | In 'meeting demands' or in 'meeting demand-associated shortfalls?' | This is corrected |
| Page 12  Line 271-272 | What does this mean? | This is corrected |
| Page 12  Line 272 | Most what? | This is corrected (facilities) |
| Page 13  Line 296 | ‘new’ media | Changed to ‘social’ media |
| Page 14  Line 315-316 | How were these coordinated to ensure that COVID-19 spread was not intensified? | Coordinating structures were set at different levels: federal, regional, zonal, woreda and facility levels(district). |
| Page 15-16  Line 353-354 | What is the total tested? | We do not have information on how many samples were tested with the help of health extension workers. However, we have included total number of samples tested at that period of time. |
| **Discussion:** Page 16, 1^st^ paragraph  Line 353-354 | I am not sure if this multisectoral team is complete without animal health and the environment as I pointed out in the result. several animal species were infected in the West, and possibly in Africa though we did not check for animal status in most part of Africa but there were some animal infection in South Africa too. Hence, I will expect that in the minimum, those two sectors should be involved. | This is already answered on page 8. |
| Page 19  Line 435-436 | But there is a caveat here that needs to be addressed. Most decentralized governments are starved of funds at the very local or subnational levels (3rd administrative levels) as is evident in many countries like Kenya, Uganda etc. How do you ensure that they have the resources enough to prosecute their duties? This cannot be assumed as a given. | Clarified: This might be related to limited experience in exercising decentralization in Ethiopia and lack of mechanisms to ensure continuous financial support. |
| Page 19  Line 436 | Why was it not sustained? | It is difficult to speculate why. However here is what we inserted: Furthermore, influences from international and national trends could operate that require further investigation |
| Page 20  Line 457-459 | How do they prevent the spread of COVID-19 during peak infection? | They are part of the health center team supported by woreda health offices. The activities are integrated with covid-response teams such as the RRT and health post-health center activities. It’s described in the subsequent statements. This is also described in the results section |
| Page 22  Line 495-497 | What of the standards? Does this meet the standards expected? Does it contain the HEPA filter? | Cloth masks are not the best option but provide some protections and is environment friendly. However, reusable facemasks vary in quality and may not be the best option. Additional reference included |
| Page 22  Line 502 | I have commented on this severally? Why call it new media? | Changed to social media |
| **Conclusion:** Page 23  Line 515 | It would have been good to have a timeline/epicurve of the outbreak situation in Ethiopia against the timeline for the waning response/case management. | Peaked in August 2020 and then declined and remained almost flat until Jan 2021 and then peaked in February 2021. Although interesting, we find it difficult to incorporate as it needs sound explanations. |
